# How Does Biological Sex Impact Mucosal Bacterial Infection? Mucosal Defenses and Bacterial Detection

**DOI:** 10.1111/imr.70112

**Published:** 2026-02-15

**Authors:** Laura Ramirez Finn, Molly A. Ingersoll

**Affiliations:** ^1^ Mucosal Inflammation and Immunity Team Université Paris Cité, CNRS, Inserm, Institut Cochin Paris France; ^2^ Department of Immunology Institut Pasteur Paris France

**Keywords:** bacterial infections, early immune detection, epithelial invasion, mucus, sex differences

## Abstract

Urinary tract infections possess substantial sex disparities in the incidence, immune response, and progression of infection. Some of these distinctions may be due to sexual dimorphism in mucosal barriers or sex differences in the initial immune response to infection. Mucosal organs are protected by a mucus barrier, however, there is little knowledge of the impact of biological sex on this layer in homeostasis and infection. Notably, despite the incidence of infection, there is a paucity of even fundamental research on bladder mucus in homeostasis and infectious disease. When bacteria encounter mucosal epithelia, they must bind and potentially invade these surfaces to initiate an infection. Whether differences in mucosal epithelia have an impact on bacterial‐epithelial interactions between the sexes is not known. When bacteria are sensed by the host, they initiate transcription factor activation, which may differ by sex of the host. Finally, sex steroid hormone receptor signaling likely also impacts innate immunity between the sexes, leading to the divergence between the sexes observed in mucosa infection.

## 1 Introduction

### 1.1 The Importance of Sex in Mucosal Immunity Research

Disease incidence, immunity, outcome, and response to treatment differ between the sexes [1]. Women tend to experience more autoimmune diseases, such as rheumatoid arthritis, multiple sclerosis, and systemic lupus erythematosus, in which the incidence is seven times greater than in men [2]. Men present with higher prevalence of infectious diseases such as hepatitis B, Legionnaire's disease, and *Campylobacter* infections [3]. In infection, men tend to have more severe disease. In some cases, men readily develop chronicity, such as in *Helicobacter pylori* infections, which leads to severe inflammation and increased likelihood of developing gastric cancer [4, 5]. Finally, at least eight FDA‐approved drugs have been removed from the market due to distinctly worse adverse effects in women, including death, compared to men [6, 7, 8, 9]. Despite these wide‐ranging associations with biological sex, integration of both sexes in experimental approaches is limited, with in vivo experiments often favoring male organisms across many fields of biological research [10]. Additionally, all too often sex is not even mentioned, including in in vitro experiments, which fail to specify the sex of cultured or primary cells [11, 12, 13]. Male bias in research is attributed to variations in steroid hormone levels throughout the menstrual cycle and behavioral differences. This justification loses strength when it is appreciated that in male organisms testosterone fluctuates, as well, and a recent meta‐analysis found that behavioral patterns are more variable in male rodents compared to female rodents [14, 15, 16, 17, 18]. Indeed, sex steroid hormones are not limited to estrogen and testosterone. All sex steroid hormones are produced in female and male organisms, and their regulation over a 24 h or monthly cycle is highly complex and inter‐regulated [19].

Sex chromosomes and steroid hormones are key drivers of sex differences in homeostasis and disease. Sex steroid hormones regulate homeostatic functions and immunity in the whole organism, as hormone receptors are found throughout the body, including on immune cells [19, 20]. Sexual dimorphism, including diverse anatomical differences, such as neuronal networks in the brain, β‐cells in the pancreas, and conducting airways in the lungs, also contribute to divergent health and malignancy between female and male organisms [21, 22, 23]. Therefore, including sex as a variable in fundamental and clinical research is essential to fully understand health and disease progression and whether treating diseases in a sex‐specific way would benefit everyone.

One approach to understand the influence of biological sex is to study a disease that is profoundly sex‐biased. Our laboratory undertook this approach several years ago to understand why urinary tract infections (UTI) are much more common in adult premenopausal women compared to men of the same age [4]. We optimized an animal model, in which female and male mice are intravesically instilled with uropathogens via a catheter placed in the urethra [24]. Notably, this method was reported to be unachievable in male mice [25, 26, 27], however, it is an approach commonly used for the study of bacterial prostatitis [28, 29]. Our initial finding, that female mice resolve their infection, whereas male mice develop chronic UTI, reflects outcomes routinely seen in the clinic, providing the justification to investigate mechanisms underlying this observation [30]. We found that female and male mice are colonized by uropathogenic *Escherichia coli* (UPEC) to the same extent initially, but male mice, and female mice treated with testosterone or dihydroxy‐testosterone (DHT), remain chronically infected. This is due to a more robust IL‐17‐mediated pro‐inflammatory innate immune response that peaks 24 h post‐infection in female mice compared to male mice [30].

Our data support that outcomes in UTI are determined by early events occurring in the first 24 h of infection. To understand these differences, we study very early infection, focusing on the first layers of defense that bacteria encounter when they colonize a mucosal surface. For example, even before bacteria reach the luminal‐facing urothelial surface of the bladder, they encounter a mucus layer that protects the underlying urothelium. Given the paucity of information about the mucus layer in the bladder, and considering other mucosal organs also present sex differences in their immune response to associated diseases, we consider the impact of sex on the mucosa in the gut and lung, and identify areas requiring further research to understand how biological sex impacts homeostasis and disease in this critical barrier site in the bladder. We also consider whether sexually dimorphic characteristics of mucosal tissue may differ, which would potentially impact bacterial interactions downstream detection by the immune system between the sexes.

## 2 Are Mucus Layers Sexually Dimorphic?

Infection is common at mucosal surfaces, such as the lung, gut, or bladder. Sex bias, in the form of prevalence, host response, or outcomes are evident in many of these infections [1, 31, 32]. While studies show that immune cells respond differently to infection at mucosal sites [1], much less attention has been given to whether barriers themselves are fundamentally sexually dimorphic impacting divergent mucosal immune responses.

### 2.1 Composition and Function of Mucus Layers

The mucus layer is a complex gel‐like layer found on the luminal surface of mucosal organs, such as the respiratory tract, gastrointestinal tract, reproductive tract, urinary tract, and the surface of the eye [33, 34]. The mucus layer covers the epithelium of an organ, lubricating the surface and providing a barrier to physical, chemical, or microbial factors, including pathogens [35]. Indeed, the mucus layer is considered an innate or classically passive immune barrier to protect from infection. It can also be a permeable layer depending on location, such as for the diffusion and exchange of gases in the lung and eye, or nutrients in the gastrointestinal tract [36, 37].

**FIGURE 1:**
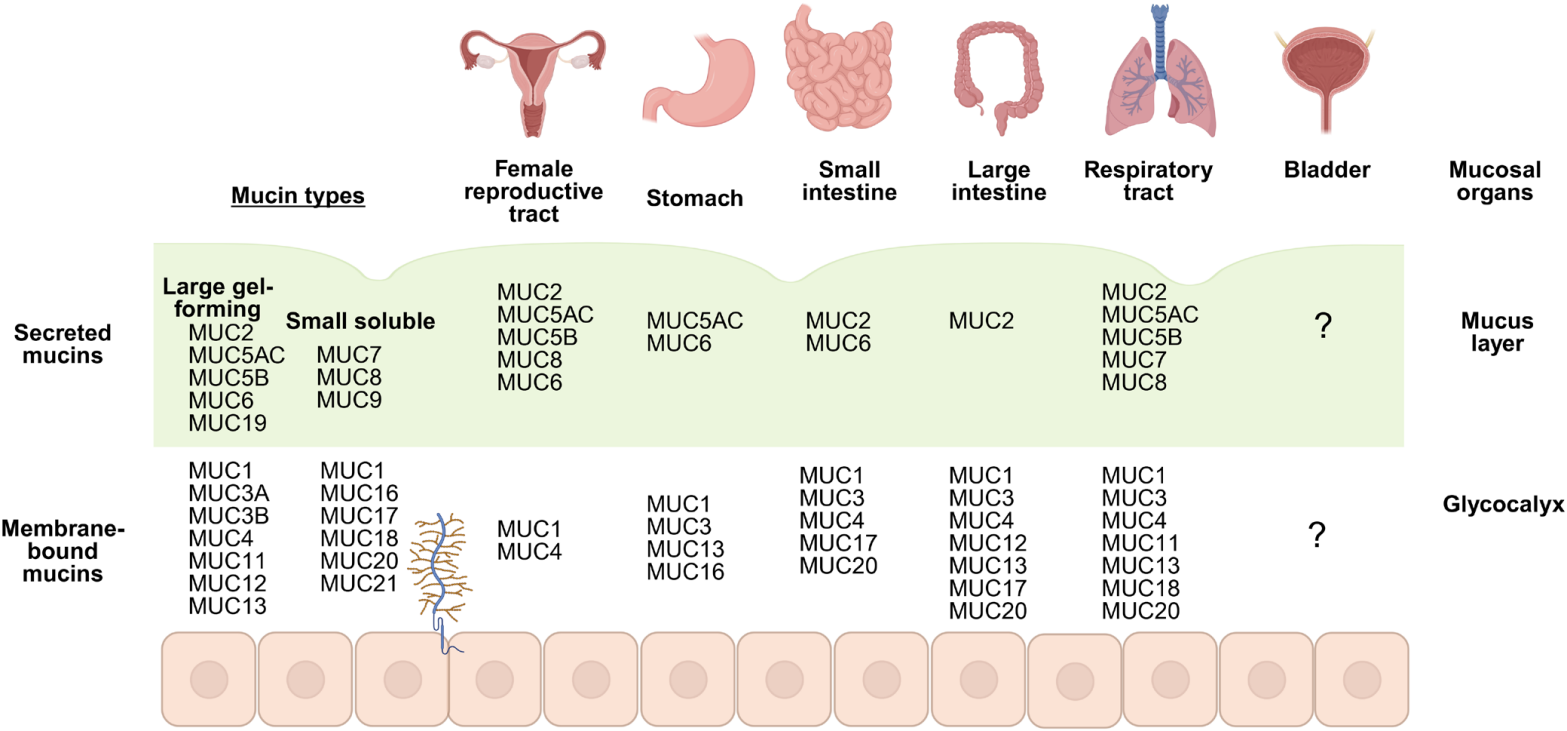
Mucin types and their unique composition in the mucus layer and glycocalyx of key mucosal organs. There are two types of mucins: secreted mucins, which can be divided into large gel‐forming and small soluble mucins. Secreted mucins make up the gel‐like coating above an epithelial layer. Transmembrane mucins form a glycocalyx, which is attached to epithelial cells. Each mucosal organ has a particular mucin composition, which forms a barrier and is tailored to the function of the organ. The schematic shows the composition of the female reproductive tract (endocervix, ectocervix, cervix, fallopian tubes, uterus, vagina, placenta, and endometrium), the gastrointestinal tract (stomach, small intestine, and large intestine), the respiratory tract, and the bladder. Assembled from [40, 41, 42, 43, 44, 45, 46, 47, 48, 49, 50, 51, 52, 53, 54, 55, 56, 57, 58, 59, 60, 61, 62, 63]. Figure created with Biorender.com.

The major building blocks of the mucus layer are heavily glycosylated glycoproteins called mucins. Glycosylation patterns include *N*‐glycosylation, *C*‐mannosylation, and *O*‐glycosylation, and these vary among mucin types. Mucins are encoded by MUC genes, and their regulation governs different forms of the expressed proteins. Twenty mucin genes exist in humans and 18 are found in mice [38]. Mucins impart biophysical properties, such as viscoelasticity and rheology, and chemical properties, such as pH, charge, ionic strength, and pore size [36, 39]. Additional complexity can be found in the type of mucin. Membrane‐bound mucins form a glycocalyx, and secreted mucins form a gel‐like layer on the glycocalyx governed by polymeric conformations of the mucins in specific organs (Figure 1).

Transmembrane mucins contribute to the homeostasis of a mucosal surface by preventing the adhesion of cells, pathogens, or debris to an epithelium [64]. The steric hindrance of these proteins, due to their large protein core, *O*‐glycosylation, charge repulsion, and hydration, makes them relatively rigid structures [35, 65]. As they can extend from the epithelium at a considerable length (e.g., MUC1 can be 200 nm long), they prevent the adhesion of gel mucins or pathogens to epithelial cells [66, 67]. Their functions are less‐well studied compared to secreted mucins but includes signaling to the interior of a cell. Signaling via their intracellular tail regulates inflammation, cell–cell interactions, cell differentiation, and apoptosis. These functions are better studied in the context of cancer compared to infection [68].

Secreted mucins form the classical gel layer that provides protection to a mucosal surface, preventing microbes from having close contact with the epithelium and maintaining immune homeostasis [69, 70]. Small soluble mucins, which are part of secreted mucins, are found as monomers because they lack cysteine‐rich domains and do not multimerize like large gel‐forming mucins. Their functions are not well studied, but MUC7 has antimicrobicidal activity in the salivary gland [71].

Differences in mucin composition at distinct mucosal sites give rise to divergent properties of mucus layers and glycocalyx [72] (Figure 1). Mucin glycosylation patterns also vary between organs, or even within the same organ, giving rise to specialized functions affecting the elasticity and rheology of a mucus layer [73]. For example, from the ileum to the rectum, there is increased fucosylation and sialylation. This results in greater acidity along the tract, which influences the transport of water and electrolytes, as well as bacterial colonization [74]. Remarkably, despite the fundamental layer of protection mucus provides, it is poorly understood whether the composition, glycosylation, or structure is different under homeostatic or malignant conditions between the sexes. Whether putative sex differences in mucins or mucus impact infection across mucosal organs is also unknown.

### 2.2 The Gastrointestinal Tract Mucus

The main function of the gastrointestinal tract is to digest and absorb nutrients and eliminate waste. One of the reasons this organ digests food and not itself, and is not constantly inflamed despite harboring a dense and diverse microbiota, in homeostasis is the presence of its mucus layer. This mucus layer varies throughout the length of the gastrointestinal tract (Figure 1). The stomach has a two layer mucus composed predominantly of MUC5AC to protect against hydrochloric acid and pepsin secreted during digestion [75, 76, 77]. The small intestine has a single loose mucus layer composed primarily of MUC2 [78]. The colon, which is the most widely studied, contains a 2‐layer mucus predominantly made up of MUC2. One layer is a dense inner layer, which is essentially void of microbes [79], and the other is a loose viscous outer layer where bacterial populations are present [70]. The inner mucus layer prevents bacterial infiltration as fluorescent beads (0.5–2 μM) cannot penetrate this layer [80]. The colon's ~10^14^ commensal bacteria are found in the loose outer mucus layer where some bacteria even derive nutrients from MUC2 and ingested polysaccharides [81, 82]. Notably, mucus‐residing bacteria produce short chain fatty acids essential for epithelial homeostasis, goblet cell differentiation via acetate, and increased sialic acid residues on mucins [83, 84]. In addition to a microbial community, secreted molecules from enterocytes, ions, water, and detached cells from intestinal epithelial cell turnover, goblet cell‐derived products (e.g., Clca3, Facgbp, Agr2) and secretory IgA are found in the outer mucus layer [70, 85, 86].

The mucus layer of the small intestine resembles the outer mucus layer of the colon except that it contains fewer commensal bacteria. This is due to mucus flushing, fluid secretion, and high concentrations of secreted antimicrobial peptides. Antimicrobial peptides are detected mainly in the mucus layer covering the small intestine, rather than the luminal space, to prevent bacteria from reaching the epithelium [87, 88]. The differences in these layers allow for specification based on function and location of the microbiota.

In addition to the mucus layers of the small and large intestine, there is also a glycocalyx formed of heavily glycosylated mucins on the surface of gut epithelial cells. MUC17 is the most abundant transmembrane mucin on enterocytes in the small intestine, with lower levels expressed in the colon [89]. This dense layer of glycans is another barrier that protects epithelial cells by preventing bacterial penetration, but can also, conversely, provide sites of interaction with glycan‐binding proteins of bacteria or bacteria themselves.

#### 2.2.1 Sex Differences in the Gastrointestinal Tract Mucus Layer

##### 2.2.1.1 Function in Homeostasis and Disease

Mucus layers throughout the intestine have a particular composition and structure to accommodate specific functions: digestion in the stomach, nutrient absorption, maintaining a sterile environment in the small intestine, and balancing the habitat of commensals without epithelial colonization in the colon. However, fundamental studies defining the mucus layer in the gastrointestinal tract have either solely focused on one sex, do not specify the sex of the sample, or when including both sexes, make no comparisons (Table 1). Thus, it is unknown whether differences in the composition and function of this layer, which may impact homeostasis or disease susceptibility between the sexes, exist. Indeed, there is little information about whether the mucus layer composition is different between the sexes, with respect to mucins, lipids, salts, or water content, which could confer distinct viscosity or rheology to these layers.

**TABLE 1:** Is sex considered in studies of the mucus layer in the gastrointestinal tract?

Site in the gastrointestinal tract	Sample type	Sex of sample	References
Stomach	Sprague Dawley rat	Male	[77]
	Rat	Male or female	[90]
	Human	Unspecified	[75]
	Human	Unspecified	[76]
	129/Svj mouse	Female	[91]
	129/Svj mouse	Age and sex matched, unspecified	[92]
	129/Svj mouse (Muc1^−/−^)	Female	[93]
Small intestine	Rat	Male	[94]
	Syrian hamster kidney BFK‐21 cells, human colorectal cancer HT‐29 cells	Cells from unsexed hamsters and cells from a woman	[95]
	C3H/HeN mouse	Unspecified	[87]
	Germ‐free mouse	Male	[81]
	Germ‐free NMRI mouse	Male	[96]
Colon	C57BL6J mouse (Muc2^−/−^)	Unspecified	[70]
	Winstar rat	Male	[97]
	Human	Unspecified	[98]
	Human	Unspecified	[78]
	C57BL/6 mouse and human	Unspecified	[85]
	C57BL6J mouse	Unspecified	[99]
	Conventional and germ‐free rat	Both sexes, no sex comparison	[100]
	C57BL6J mouse	Unspecified	[101]
	Gnotobiotic mouse	Both sexes	[102]
	RGF rat	Male	[84]
Entire tract	Rat	Male	[103]
	C57BL6 mouse	Both sexes, pooled	[79]
	Human	Both sexes, only compared at the level of the reproductive tract	[89]
	C57BL6 mouse	Unspecified	[88]
	C57BL6 mouse	Both sexes, compared	[104]

Fundamental research on the composition and function of the gastrointestinal mucus layer is stated and specifies what part of the tract is studied, what species is used, and whether the sex is reported.

Studies that specifically compared the gastrointestinal mucus layer have identified isolated differences between the sexes. For example, the thickness of the mucus layer is similar in the stomach of rats [105]. The thickness of the mucus layer in the colon decreases with age in both sexes due to bacterial interaction with the epithelium [106]. While this study showed that *Muc1* expression decreases with age in male colonic mucus and *Muc6* increases with age in females [106], it lacks sufficient convincing evidence to support the claim that the mucus changes substantially between the sexes and over time. Despite comparing from young mice to aged mice within the sexes, the work rarely compared young females and young males to each other in homeostatic conditions [106]. Increased mucus thickness in the colon of female mice compared to males was also observed, but this is shown as mucus density in the villi, rather than mucus height [107], which is a less robust measurement. Thus, there are still significant gaps in our knowledge regarding mucus thickness in the rest of the gastrointestinal tract and in other species, such as humans.

In wild mice from the Isle of May, female mice have increased mucin sialylation in their proximal colon compared to wild male mice. Interestingly, this difference is enhanced in laboratory C57BL/6 mice compared to wild mice and is regulated by ERα signaling [108]. This is important because sialylation confers charge to mucins and prevents their enzymatic breakdown [109]. Therefore, sialylation enhances barrier function and impacts the ability of bacteria to pass through the mucus [110], suggesting bacterial invasion may differ between the sexes.

In the pathological condition of stroke, female mice have increased *Muc4* and *Muc2* expression in the colon, mediated by estradiol, whereas male mice do not increase mucin production. This leads to a thicker colonic mucus layer in female mice compared to a disrupted layer in male mice that allows the microbiota to encroach upon the epithelium and cause exacerbated neuroinflammation [111]. Cultured human female intestinal epithelial cells also increase mucin production in response to estradiol therapy, leading to increased viscosity and elasticity of the mucus [112]. This suggests there are sex‐specific responses in pathological contexts at the level of the mucus. As estrogen may regulate mucin thickness, and estrogens vary throughout life, the barrier function of mucus and its properties may also fluctuate over time. Additionally, whether estrogen also regulates mucus in male organisms is not known, although mucosal‐associated stromal and immune cells express estrogen receptors to varying degrees [113]. For example, estrogen receptor beta (ERβ) is more highly expressed in female mouse goblet cells in the conjunctiva and estrogen receptor alpha (ERα) localizes to the nucleus only in female goblet cells, whereas the androgen receptor (AR) is only expressed in male goblet cells [114]. This indicates the goblet cells may also have differential functions between the sexes.

Maintenance of the mucus layer in the gastrointestinal tract is a tightly regulated process that is essential for its proper functioning. When mucus production is dysregulated, it can lead to auto‐inflammatory conditions such as inflammatory bowel disease, including Crohn's disease and ulcerative colitis [115]. Patients with inflammatory bowel disease have dysregulated thickness of their intestinal mucus [116, 117], which brings the microbiota closer to the epithelium, initiating an inflammatory response that damages the tissue. Key for mucus production is different populations of goblet cells. Flavin‐containing monooxygenase‐5 is important for intestinal homeostasis, as its absence leads to disrupted crypt structures and goblet cell location only in female mice [118]. This suggests that female and male goblet cells are regulated differently and may have diverse functions. The altered goblet cell location leads to a thinner mucus layer, which would be predicted to allow microbiota encroachment on the epithelium [118]. As the microbiota approaches the epithelium, this increases the possibility of leaky gut syndrome and inflammatory bowel disease. Interestingly, women have increased prevalence of Crohn's disease [119, 120]. These findings highlight the need to study homeostatic function in both sexes as they may reveal discreet mucus functions between the sexes, which can then be translated into disease prevalence, pathogenesis, or treatment [121].

MUC2, the main component of the gastrointestinal mucus, is crucial for the protection of the gut surface. Mice deficient in Muc2 have bacteria in direct contact with the colonic epithelium and develop colitis [69, 85]. Similarly, samples from patients with ulcerative colitis also have bacterial association with the epithelium, lower levels of MUC2 and secretory goblet cells, and in a subset of patients, increased penetrability of their mucus layer [122, 123]. Unfortunately, the impact of biological sex is not clear, as the preclinical studies were performed only in male mice [69, 85]. In the human studies, only 7 out of the 28 patient samples were women [123] or the sex of the donors was not specified [122], demonstrating a missed opportunity to understand why inflammatory bowel disease is more common in women.

The microbiota is an essential component of the mucus layer in the gastrointestinal tract and is made up of a variety of bacteria, viruses, archaea, and fungi. These microbes are essential to maintain homeostasis, helping to regulate immune function and metabolism [124]. Notably, the gut microbiota of women and men is different throughout life [125]. Male mice have higher levels of fecal microbiota than females, but female mice have greater variation of their microbiota composition throughout the day compared to males [126]. Quite interestingly, deletion of the clock gene BMal1 abrogates observed differences in microbial composition between female and male mice [126]. The gut microbiota may also contribute to regulation of androgens, as germ‐free female mice have higher levels of testosterone compared to their specific pathogen free (SPF) counterparts, and male germ‐free mice have lower levels of testosterone than male SPF mice [127]. Male mice have a higher abundance of microbial phyla than female mice. This phenotype is lost when male mice are castrated and can be restored with exogenous DHT [128].

In infants, delivery method, feeding, and the environment can all impact microbiota composition. Interestingly, baby girls born vaginally have higher alpha‐diversity than boys, who have lower abundance of *Firmicutes* and greater abundance of *Proteobacteria* [129]. This shows that despite the same route of birth, fundamentally, biological sex can impact the composition of the microbiota from infancy [129]. The impact of sex on the microbiota continues based on feeding habits, and into adulthood and elder years, with men presenting higher levels of *Bacteroides* and *Prevotella* [130, 131]. Similarly, aged male mice have a decrease in beneficial *Lactobacillus spp*. and unclassified *Clostridiales*, whereas pathobionts are increased. This finding suggests that gastrointestinal diseases may be related to the thickness of the mucus in older individuals [106].

Thus, while much remains to be investigated, the mucus layer quite likely differs between the sexes in terms of composition and glycosylation, which would impact its function between the sexes. It also differs in terms of the microbiota, which could enhance differences in homeostatic functions, such as thickness of the layer, production of short chain fatty acids, immune tolerance, and defense against pathogens. Simply using samples or organisms from both sexes in studies of the gastrointestinal tract would contribute substantially to expanding our understanding of how biological sex shapes this mucosal surface.

#### 2.2.2 Sexually Distinct Bacterial Gastrointestinal Infections Are Poorly Understood

Despite the protective function of the mucus layer, pathogens still bypass it to reach the epithelium. Bacteria have different mechanisms to do so, tailored to the site of infection.

##### 2.2.2.1 *Helicobacter pylori* in the Stomach

*Helicobacter pylori* infect the stomach and can consequently cause gastric ulcers and cancer [132]. To do so, *H. pylori* must survive the acidic environment of the stomach and penetrate the two‐layer mucus lining the epithelium. To neutralize stomach pH, the bacteria produce urease to elevate NH_3_ and CO_2_ [132]. *H. pylori* modulates the pH of the mucus itself, which decreases the viscoelasticity of the layer, facilitating bacterial mobility through the mucus [133, 134]. Once through the soluble mucus layer, *H. pylori* reach *the* glycocalyx on the epithelial surface. *H. pylori* binds MUC1, the most abundant transmembrane mucin in the stomach, via two adhesins [135]. When *H. pylori* bind MUC1, the extracellular domain of MUC1 is shed from epithelial cells as a protective mechanism. During infection, MUC1 is, therefore, removed from the epithelial surface allowing bacterial attachment.

*H. pylori* infections have a prominent sex difference with greater infection in male children and adults [31]. However, the reasons underlying this bias are not known. While the pH of the stomach is similar between the sexes [136], it may be that chemical properties or the composition of the layers are divergent between the sexes. Despite more men experiencing this infection, many studies have used female cells and mice [91] or human gastric juice [91] and porcine gastric mucus [133] of unidentified sex of origin.

##### 2.2.2.2 Salmonella Infection in the Small and Large Intestine

The small and large intestine present different environmental challenges for pathogens to reach the epithelium. Bacteria need to overcome the flow of intestinal contents and becoming trapped in the mucus. *Salmonella Typhimurium* moves chemotactically towards the epithelium when in the mucus using flagella [137, 138, 139]. *S. typhimurium* preferentially invades the cecum, as this section of the intestine lacks the thickness of the two layer mucus, and preferentially invades epithelial cells on the surface that are poorly covered by mucus [140]. This cecal invasion of the bacteria elicits an interferon 𝛾 response that leads to the loss of Muc2 vacuoles in goblet cells [141].

*Salmonella* infection incidence is greater in post‐pubertal women compared to men, whereas it is higher in male children up to the age of 15 years [142], suggesting sex steroid hormone signaling may contribute to disease susceptibility. Despite this, the studies cited above used only female or male mice [138, 139], both sexes pooled together [143, 144], or both sexes were used but randomly assigned to different experimental groups [140]. Studying the interaction of *S. typhimurium* with the mucus may give clues as to differences between the sexes and different mucin composition, glycosylation, or microbiota‐derived byproducts of the mucus may impact the pathogenesis between the sexes.

### 2.3 The Respiratory Tract Mucus

The respiratory tract is divided into the upper respiratory tract: nose, pharynx, larynx; and the lower respiratory tract: trachea, proximal bronchi, and lungs. The respiratory tract is also a mucosal surface in contact with the external environment and in need of protection against inhaled particles and pathogens [145].

The respiratory mucus is organized in a gel‐like layer formed of secreted mucins, similar to the gastrointestinal tract (Figure 1). The transmembrane mucin glycocalyx is found in the periciliary space. Secreted mucins are above the periciliary space in a layer. Human MUC5AC and MUC5B are the most abundant mucins in the mucus gel and can be found in the sputum of healthy individuals in similar quantities [146, 147]. MUC5AC and MUC5B are homotypic polymers that form long polymeric chains, which can then be entangled into a mesh by noncovalent cross‐linking [148]. MUC5AC is produced by goblet cells, whereas MUC5B is produced by surface secretory cells and by mucous cells of the submucosal glands [149, 150]. As these cell types have distinct locations, this impacts mucin localization in the lung. Homeostatic mouse airways resemble the distal human airway with bronchi and bronchiole less than 2 mM in diameter. Mice have little to no Muc5ac [151, 152], and Muc5b is found in airway secretory cells. Instead, MUC1, MUC4, and MUC16 cover the surface of epithelial cells in the periciliary space forming a viscous glycocalyx [149, 153]. MUC1 and MUC4 localize to the bronchi and bronchioles [154, 155]. The mucus layer forms a motile barrier, as beating cilia contribute to mucociliary clearance. This mucus movement helps clear pathogens, apoptotic cells, and other debris to maintain proper lung function [156].

#### 2.3.1 Sex Differences in the Respiratory Tract in Homeostasis and Disease

No comparisons of homeostatic composition or function of the mucus layer have been made between the sexes in the respiratory tract (Table 2). This is striking considering that lung diseases, such as asthma, display pronounced sex differences [166]. Interestingly, asthma is more common in prepubescent boys compared to girls; however, asthma incidence increases in women compared to men after puberty [167, 168, 169]. One reason for this is that women and men have anatomically distinct airways, in which women have decreased airway diameter and lung size [170, 171]. This could contribute to reduced lung function in women, exacerbating respiratory symptoms [172].

**TABLE 2:** Studies performed to understand composition and function of the mucus in the respiratory tract.

Sample type	Sex of sample	References
Human	Unspecified	[146]
Human	Both sexes, no sex comparison	[147]
Human	Unspecified	[157]
Human	Unspecified	[158]
Human and SW403 cells	Human unspecified and cells of female origin	[159]
Human	Unspecified	[160]
Rat	Male	[161]
NCIPH292 cells and Fischer rats	Cells of female origin and male rats	[162]
Human	Unspecified	[163]
Human	Unspecified	[164]
Human	Unspecified	[165]
A549 human cells	Male	[43]
Mice	Both sexes, but no sex comparison	[154]
Mice	Unspecified	[155]
Human	Unspecified	[149]
Mice	Unspecified	[150]
Fischer rats	Male	[151]
Mice	Female	[152]

*Note:* The table specifies the type of sample used and the sex of this in each study on respiratory tract mucus.

The prevalence of chronic obstructive pulmonary disease and cystic fibrosis, respiratory diseases characterized by increased mucus production, also varies by sex. Whereas chronic obstructive pulmonary disease was largely thought to be a male‐dominant disease due to smoking habits, it is actually found in higher numbers in women [173].

#### 2.3.2 Sex Differences in Respiratory Bacterial Infections and Mucus Interactions

While mucociliary clearance mechanisms help fight infection, pathogens still manage to hijack components of the mucus layer, leading to successful colonization.

*Pseudomonas aeruginosa* is a common causative agent of pneumonia. Men are more susceptible to respiratory tract infections with increased morbidity and mortality associated with infection [32, 174]. *P. aeruginosa* uses mucins in the lung to establish infection. Its flagellin bind MUC1, based on the level of glycosylation, and initiate downstream signaling [175, 176, 177, 178]. Mice lacking Muc1 clear the infection more efficiently, suggesting an antibacterial role for this mucin in infection [179, 180, 181]. Notably, *P. aeruginosa* can bind to the epidermal growth factor receptor (EGFR) with higher affinity than its classical ligand and with its activation, induce increased expression of *Muc5ac* [161].

Despite this mechanism, investigation of mucins and *P. aeruginosa* interactions either do not mention the sex used for the mouse model [161, 179, 181] or use only female models [176, 180]. Interestingly, in an in vitro approach to test the adhesion of *P. aeruginosa* to sialic acid, bacteria bind Mz‐Ch cells better than three other cell lines, suggesting the cells possess different degrees of sialylation [178]. Of note, the three cell lines with lower binding, AF549, HepG2, Caco‐2, are from male lung, liver, or gut cells, whereas Mz‐Ch cells are from female biliary epithelial cells [178]. This means sialylation may differ between the sexes, which would be predicted to impact bacterial binding or attachment to establish consequent infection, leading to different outcomes between the sexes.

*P. aeruginosa* is also a common opportunist pathogen in patients with cystic fibrosis [182], a genetic disease in which the cystic fibrosis conductance regulator protein is mutated, leading to decreased expression or activity of a chloride channel on cell membranes [183]. In the lungs, this mutation contributes to a highly viscoelastic mucus layer [184]. The thickness of the mucus and the reduction of the airway surface liquid layer cause the mucus layer to attach to the epithelium. This creates an environment bacteria thrive in as the antimicrobial peptides are ineffective [185]. The thickened mucus also impairs the beating of cilia, preventing mucociliary clearance and forming mucus plaques [185]. Mucus plaques can generate areas in the lung with lower oxygen or even anaerobic conditions that favor the persistence of *P. aeruginosa* [186].

Women have more severe comorbidities with cystic fibrosis than men, leading to higher rates of mortality [187]. Girls with cystic fibrosis have lower survival rates than boys, with an increased decline in lung function. This could indicate the involvement of hormones in increasing life‐threatening symptoms. For example, 17‐β‐estradiol reduces airway surface liquid, and in cystic fibrosis, this leads to a mucus layer attached to the epithelium [188]. Estrogen can indirectly impact chloride export, resulting in a thicker mucus layer [189]. Estradiol also increases the overproduction of MUC5B, which may enhance the formation of mucus plaques in women [190]. In healthy airways, progesterone decreases the frequency of cilia beating, whereas estradiol has an opposite effect, promoting increased beating of cilia [191]. In cystic fibrosis, however, estradiol slows the beating of cilia, which prevents the clearance of mucus and mucus plaques containing bacteria [192]. Not only does estrogen impact the physiological functions of the protective mucus layer in cystic fibrosis, it also enhances the virulence of *P. aeruginosa* . Estradiol can induce the pathogenic mucoid form of *P. aeruginosa* in vitro, and indeed these forms have been isolated from patients during their follicular phase, when estrogen rises [193]. Treatment of bronchial epithelial cells with estradiol enhances the secretion of *P. aeruginosa* virulence factors, bacterial movement that enhances attachment to epithelial cells, and the formation of biofilms [192].

*P. aeruginosa* interacts with the mucus layer to attach and invade the underlying epithelium. As attachment and invasion would be impacted by mucus layer properties and composition, understanding homeostatic differences or the effect of hormones on the mucus would also bring new knowledge about bacterial behavior and infection. Thus, the study of one of the most common nosocomial infections, with distinct prevalence between the sexes in healthy individuals and cystic fibrosis patients, would benefit from the inclusion of sex as a biological variable.

### 2.4 The Neglected Bladder Mucosa

Studies of the bladder are significantly behind those of other mucosal organs, despite that it is a common site of infection and a key barrier tissue. Very little is understood with respect to its mucus layer, which remains somewhat controversial.

#### 2.4.1 Are There Sex Differences in the Bladder Mucus Layer?

The bladder is another mucosal organ contiguous with the external environment and thus requires a myriad of host defense mechanisms to protect against pathogens and create a tight barrier against metabolic waste in urine [194]. The epithelium of the bladder, the urothelium, is made of three to seven layers of epithelial cells, depending on the organism, that change morphologically upon extension and contraction of the bladder. Strong tight junctions between urothelial cells and formation of uroplakin plaques on the surface of urothelial cells help maintain the impermeable surface [195].

The urothelium has a layer of protective mucus and classically, this is described as a dense glycosaminoglycan (GAG) layer, formed of hyaluronic acid, chondroitin sulphate, and proteoglycans [196]. A thin layer of hyaluronic acid can be observed above the urothelium in the bladder of rabbits and humans [197] and this is referred to as the “mucopolysaccharides” of the bladder. Scanning electron microscopy reveals a thin layer of polysaccharides in rat bladders treated with anti‐mucus serum (antibodies raised against mucus scraped from rat bladders) [198]. Suggesting that there is a thinner mucus layer in the bladder compared to the intestine, human bladders reconstructed from the ileum have a very thick continuous layer compared to healthy pig bladders [199]. Additionally, the urine content of mucins is 30 times higher in patients with ileum‐reconstructed bladders than healthy control individuals [199].

In rabbit bladders of unspecified sex, sialylated glycoproteins are detected at greater levels than GAG and this corresponds to MUC1 [200, 201]. In the bladder of male donkeys, there are sialoglycoproteins from umbrella cells and these vary throughout the tissue, suggesting these are mucin‐type glycans [202]. These glycans appear to be different among species as well, including rat, rabbit, and male pig bladders [200, 203, 204]. In male pig bladders, the glycans are most likely scaffold support for MUC1 [204]. *O*‐glycans can be detected after trypsinization, suggesting they belong to membrane‐bound rather than secreted mucins. However, this work was performed using porcine bladders of unspecified sex [205].

The mucus layer prevents bacterial adhesion [206] and impacts tissue [207]. The GAG layer limits the accessibility of urothelial cells to pathogens and disrupting this layer with HCl, Triton X‐100, or pentachlorophenol increases bacterial entry [208]. These harsh treatments most likely damage the urothelium as well, which would be expected to facilitate bacterial entry into the tissue. Adding heparin or sodium pentosan polysulphate to the surface of acid‐treated bladders replicates GAG layer protection, preventing bacterial binding [209, 210].

In these studies, the GAG layer is also referred to as the mucin layer. However, this misuse of terminology ignores classical mucus layers; the glycocalyx of transmembrane mucins and the gel mucus formed of gel‐forming mucins, which make up the ensemble of the mucus. These layers are essential in a mucosal organ in contact with the external environment. There is a paucity of studies investigating classical mucus structure, with mucin components, glycosylation, organization and how these factors may be different between the sexes in homeostasis, or a heavily sex‐biased infection, such as UTI. In bladder studies, the sex of the human bladder biopsy samples, rabbits, and pigs was undetermined, or only female rats or male rabbits, pigs, and donkeys were used [197, 198, 200, 201, 202, 203, 204, 208, 209].

#### 2.4.2 Urinary Tract Infections and Sex Steroid Hormones

UTI are one of the most common infections world‐wide, impacting more than 400 million people every year [211]. UTI are caused almost entirely by bacteria, and both Gram‐negative and Gram‐positive bacteria can establish infection in the bladder [212]. Uropathogenic *Escherichia coli* (UPEC) is the causative agent in approximately 85% of infections in otherwise healthy individuals [213, 214, 215].

UTI disproportionately affect adult women over men; however, incidence between the sexes differs throughout life [4]. In early life, however, baby boys are more likely to experience a UTI compared to baby girls (20% vs. 8%), although the rate of recurrent infection is the same [216, 217]. The most striking difference is observed in adolescents and adults, in which pre‐menopausal women experience almost 4 times the infections than men [211, 218, 219, 220]. Later in life, the prevalence of infection decreases in elderly women and increases in men, leading to fairly equal prevalence [221]. This change in the prevalence of infection coincides with changes in steroid hormone levels, with the highest incidence of infection in women occurring when estrogens peak, and when androgens are at their highest and estrogen lowest in men [19]. Despite experiencing less frequent infection, men experience more serious UTIs that are complicated to treat and require longer treatment with antibiotics [220, 222]. Indeed, UTIs are infections that distinctly impact women and men with different incidence, prevalence, and disease outcomes. For this reason, it is important to study both sexes to better understand the different pathologies and improve treatment between the sexes.

Sex steroids are an important factor influencing infection in the bladder. In a study from our laboratory, upon observing that females resolve their infection, whereas male mice do not, we speculated that steroid hormones influence UTI outcome [30]. We found that female mice treated with testosterone can no longer clear their infection, mirroring the chronic UTI that develops in male mice. Strikingly, testosterone and dihydroxy‐testosterone administration correlate with decreased numbers of leukocytes, including γδ T cells and type 3 innate lymphoid cells, compared to control animals, which produce the pro‐inflammatory cytokine IL‐17 [30]. We also observed that castrating mice, as young as 3 weeks of age, did not confer the ability of these animals to resolve their infection. This suggests that androgens, such as testosterone or dihydroxy‐testosterone, may imprint the male immune system. It also suggests that androgens act within the bladder to modulate disease and immunity, as has also been observed in bladder cancer [4].

Given these differences in UTI, and that a small amount of evidence suggests mucus may differ between the sexes in the gastrointestinal tract, we hypothesize that the bladder also has sexually dimorphic mucus. To gain insight into how hormones may impact a bladder mucus layer, we can look to the cervicovaginal mucus and fluid (Figure 1). With the monthly cyclical changes in steroid hormones, the cervical mucus changes throughout the menstrual cycle. With increased levels of estradiol during the follicular phase, there is increased mucus, with higher MUC4 and MUC5B expression, leading to a thinning of the mucus layer, which becomes more aqueous to allow sperm entry and migration to the uterus [223, 224]. During the luteal phase, when progesterone increases, mucus decreases and becomes thicker and opaque [63]. This coincides with ovulation when the vaginal pH rises and mucus becomes more viscous and stretchy [225, 226]. It is not a large stretch to hypothesize that steroid hormone cycling may impact bladder mucus, similar to its effect on cervical mucus, changing its penetrability to uropathogens in women compared to men and at different points in the menstrual cycle [215].

The cervicovaginal mucus not only helps prevent infection but also maintains a healthy vaginal microbiota [227]. The presence of *Lactobacillus spp* in the vaginal microbiota can contribute to potent antimicrobial effects, for example by trapping higher concentrations of HIV‐1 virions [228]. Women with bacterial vaginosis have greater abundance of *Gardenella vaginalis*. This can cause increased pH and decreased mucus viscosity [63]. When female mice with dormant UPEC reservoirs in their bladder following infection are exposed to a challenge of *G. vaginalis* intravesically, this leads to the reactivation of UPEC and a UTI [229]. This suggests the vaginal microbiota interact with the bladder, although whether these interactions impact the mucus layer is entirely unknown.

During menopause, when menstruation ceases, there is a significant decrease in estrogens and progesterone [230]. The cervical mucus is decreased and becomes more viscous, with a tighter glycoprotein network [231]. The cervicovaginal fluid is less viscous, with a higher pH than in pre‐menopausal women [63]. During this time, UTI incidence increases compared to pre‐menopausal women [220]. The same change in UTI incidence is observed in men, correlating with decreases in testosterone later in life [220]. This supports that changing steroid hormonal environments may shape the mucus layer in the bladder during different phases in life between the sexes, and this may contribute to differences in UTI between the sexes.

An added layer of complexity in understanding urogenital mucus layers is the use of steroid hormonal contraception in pre‐menopausal women and hormone therapy in post‐menopausal women. Progesterone‐based contraception functions by creating a thick, viscous mucus and thick cervicovaginal fluid that prevents the migration of sperm to the uterus [63, 226, 230, 232, 233, 234]. Whether the use of hormonal contraception directly impacts infection risk is unclear. Estrogen hormone therapy in post‐menopausal women decreases vaginal pH and increases vaginal fluid [235]. Although unstudied, it is likely the increase in estrogen levels reverses the effects of menopause, leading to greater production of mucus and a more viscous layer [231]. Interestingly, the use of vaginal estrogen therapy improves urinary symptoms, such as urination frequency, urgency, and incontinence, as well as recurrent UTI [236]. Topical estrogen therapy reduces the incidence of recurrent UTI in menopausal women from 5 times a year to twice a year [237, 238, 239].

Thus, there is preliminary evidence of a glycosylated layer in the bladder of several species, but more research is needed to determine the mucin composition and the function of this in the context of infection between the sexes and how these factors impact infection risk and pathogenesis over a lifetime.

## 3 Bacterial Interactions at the Urothelial Surface

### 3.1 Bacterial Binding to Urothelium Membrane Proteins

Although mucus provides a barrier, bacteria have mechanisms to bypass or penetrate this obstacle. Once bacteria transit a mucus layer successfully, the next step is to colonize epithelial cells. Whether bacteria bind or invade epithelial cells differently between the sexes is yet to be uncovered. In the study of UTIs, this is predominantly because most studies are performed exclusively in female animals and humans [4], and surprisingly, in male cells in in vitro studies. Indeed, the sex bias in incidence has strongly influenced experimental models, leading to a large gap in our knowledge.

In UTI, UPEC use the FimH adhesin on the end of their type I pili to bind mannose on host glycoproteins on the surface of the bladder to invade urothelial cells [240, 241]. FimH binds to the glycocalyx of urothelial cells of the ureter, extracellular matrix proteins (fibronectin and laminin), antigens of the carcinoembryonic family, Tamm‐Horsfall protein, CD48, CD11, CD18, uroplakin Iα and integrins α2, β1, α3, α6, and β4 on urothelial cells [242, 243, 244, 245, 246, 247, 248, 249, 250, 251]. Although FimH binds several integrins, α3 and β1 integrin binding leads to bacterial invasion [251]. Estrogen increases the expression of UPIα and β1 integrin on exfoliated urothelial cells from premenopausal women or post‐menopausal women treated with estradiol, suggesting bacterial invasion may be enhanced in females [252]. UPEC also use FimH to bind and enter vaginal epithelial cells, alongside other unknown mechanisms, to establish reservoirs in the vagina [253]. There is an additional adhesin on pyelonephritis UPEC strains CFT073, called UpaB, which binds to extracellular matrix proteins [254, 255]. One of its binding sites interacts with glycosaminoglycans and the absence of this site leads to decreased colonization in female mouse bladders [256]. It is still unknown how GAG are involved in bacterial invasion of urothelial cells and whether they differ between the sexes.

Remarkably, many of the abovementioned studies performed their experiments using human urothelial cells from men [241], cells from the ureter with no specification of sex [242, 253], purified proteins with no mention of sex or from male samples [243, 244, 245, 246, 247, 248, 249, 250]. Some of these cell lines, such as 5637, are also advanced cancer cell lines. When comparing the entry of UPEC in the bladder compared to vaginal epithelial cells, the bladder cells were of male origin [253]. Thus, there is a great gap in our knowledge of whether bacteria bind differently to female and male urothelial cells or whether the expression of FimH ligands differs between the sexes, as this could lead to different invasion between the sexes and impact the response to UTI.

In the colon, UPEC strain UTI89 binds to the inner and outer mucus layers of the colon, as well as the epithelial surface [257]. This suggests UPEC can bind components of the mucus layer and the transmembrane glycocalyx. In the absence of FimH, UTI89 is not found in the mucus layers, suggesting this binding is necessary for retention in the intestine [257]. However, this study was only performed in female mice. Thus, the specific mucins bound by type I pili in the gut and whether they are different between the sexes, remains to be determined. Another strain of *E. coli* , O157:H7, also binds to scraped mucus from rat and human samples of undefined sex only when they express type I pili [258]. *E. coli* expressing an invasion protein from *Yersinia pseudotuberculosis* has increased β1 integrin‐mediated epithelial invasion in the intestine, when there is MUC1 intracellular signaling [259]. This may indicate UPEC also bind mucins in the bladder.

Thus, whether the expression of FimH ligands or other receptors, which signal in urothelial cells, is different between the sexes during homeostasis in the bladder is still unknown and whether there are differences in the binding of UPEC to the epithelial surface and their consequent invasion is yet to be studied in both sexes.

### 3.2 Secreted Factors Impacting Invasion and Colonization

UPEC secrete many virulence factors, some of which mediate bacterial invasion of urothelial cells. Approximately 30% of UPEC possess the gene for cytotoxic necrotizing factor 1 (*cnf1*) [260]. When CNF1 enters a eukaryotic cell, it deamidates a glutamine residue to glutamic acid in RhoGTPases [261, 262, 263]. This modification leads to a permanent activation of RhoGTPases by inhibiting their GTPase activity to convert to their inactive GDP‐bound state, thereby inducing membrane ruffling and actin stress fiber formation. These changes result in epithelial membrane disruption and passive bacterial internalization [264].

Primarily in vitro studies evaluate the effect of the toxin in male rat urothelial cells [264]. The effect of the toxin in vivo is controversial with no significant differences observed in bacterial invasion in female bladders and kidneys or in male prostates [265, 266, 267]. Similarly, infections with UPEC that possess or lack CNF1 colonize the bladder of female mice equally well [268]. By contrast, in a competitive infection scenario in female mice, UPEC deficient in CNF1 were outcompeted by a wildtype strain sufficient for CNF1 during the first week of infection. Surprisingly, in male mice, the advantage conferred by the presence of CNF1 is delayed to later stages of infection (**unpublished data**). This suggests that a bacterial toxin can have a different effect in female and male bladders, impacting the clearance of bacteria.

Another toxin secreted by 50% of UPEC is α‐hemolysin (HlyA), which induces pore formation [269, 270]. HlyA degrades paxillin, an adaptor protein between the plasma membrane and the actin cytoskeleton in urothelial cells [271]. Interestingly, *hlyA* is in the same locus as *cnf1*, together influencing the turnover of the tissue [271, 272]. The amount of HlyA in infection is important, as overexpression increases urothelial cell death and exfoliation, while reducing bacterial burden in male 5637 epithelial cells [273]. Whether this may impact female and male urothelial cells differently is unknown, as urothelial cell exfoliation and regeneration kinetics have not been directly compared between female and male mice in UTI.

In summary, it is unknown whether the initial binding and consequent entry of bacteria into urothelial cells in the bladder is different between the sexes. This will be dependent on whether transmembrane ligands that facilitate bacterial binding and internalization diverge between the sexes in homeostasis, leading to distinct intracellular signaling or modulation by bacterial toxins.

## 4 Early Initiation of the Innate Immune Response

### 4.1 Pattern Recognition Receptors

When bacteria are at a mucosal surface they will be detected by pattern recognition receptors (PRR), which initiate a downstream immune response. Several Toll‐like receptors (TLR) are present in healthy human urothelial cells of male origin [274], however, only TLR4, TLR5, and TLR11 have an impact on the response to UPEC in mice [275, 276, 277, 278]. TLR4 and TLR5 detect lipopolysaccharide (LPS) and flagellin, respectively, but there is no known ligand for TLR11 [278, 279, 280]. Cell surface TLR signaling leads to the activation of adaptor proteins MyD88 (myeloid differentiation primary response gene 88), TIRAP/Mal (TIR‐domain–containing adaptor/MyD88 adaptor–like), TICAM1/TRIF (TIR‐domain–containing adaptor molecule 1/TIR‐domain–containing adaptor‐inducing interferon β), and TRAM (TRIF‐related adaptor molecule) [281]. The downstream signaling cascade can either be MyD88‐dependent or independent and can lead to the activation of interferon‐regulatory factor (IRF), nuclear factor kappa B (NFκB), or activator protein (AP‐1) transcription factors [282, 283]. MyD88‐independent signaling also leads to the activation of NFκB and IRF3 [284]. NFκB induces transcription of pro‐inflammatory cytokines, such as IL‐1 or TNF; chemokines, adhesion molecules, cell cycle regulators, and anti‐apoptotic factors to initiate an innate immune response following bacterial detection [283, 285].

TLR4 is found on the urothelium lining the entire urinary tract, but its adaptor protein CD14 is not detected in human biopsies of unspecified sex [286, 287]. UPEC infection of urothelial cell lines shows that LPS activates TLR4 and CD14 to activate NFκB and p38 MAPK [288]. C3H/HeJ mice, which have a point mutation in the *Tlr4* gene impairing detection of LPS, have more persistent infections leading to ascending UTI [275, 276]. C3H/HeN mice, with a functional TLR4, experience a peak of neutrophil infiltration at 24 h post‐infection. Patients with asymptomatic bacteriuria have lower levels of TLR4 on their circulating neutrophils [289]. *TLR2* and *TLR4* polymorphisms are present in young girls and boys with recurrent UTI, supporting TLR sensing is important to respond to UPEC in humans [290]. The role of TLR2 in initiating a response to UPEC in mice is yet to be determined. TLR5 is expressed in both the kidney and bladder, with higher levels in the bladder [277]. Mice lacking TLR5 have similar bacterial burden until day 5 when bacterial burden increases significantly in knockout mice compared to wildtype mice [277]. The presence of TLR5 leads to a pro‐inflammatory environment, including production of CXCL2, CCL2, IL6, and TNF [277]. Human urothelium produces the cytokines IL‐6 and IL‐8 in response to TLR5 activation by flagellin, but not TLR4 activation [291]. In children with recurrent UTI and no urinary tract malformation, there are no differences between the sexes in terms of incidence of infection and polymorphisms in their *TLR2* and *TLR4* genes [290]. However, the sample size was quite limited in each specific group separated by sex in this study [290]. Finally, TLR11 is strongly expressed in the urothelium and kidney epithelium in mice [277]. TLR11‐deficient mice have the same amount of bacteria in their bladder as wildtype mice, but increased bacterial burden in their kidneys 4 days after infection [278]. Following UPEC sensing, TLR11 signals via NFκB [278]. Therefore, TLR pathways that sense UPEC are crucial for activating transcription factors that lead to the production of cytokines and chemokines during UTI.

The contribution of intracellular TLR is still to be determined in UPEC infection; however, UPEC is detected by other receptors intracellularly. NOD2 (nucleotide‐binding oligomerization domain containing 2) is an intracellular receptor that binds to muramyl dipeptide, a peptidoglycan motif of bacteria that activates NFκB [292, 293]. Despite being a potent activator of an antibacterial response in the gut [294], NOD2 does not play a role in the immune response to UPEC in the bladder [295]. The detection of bacterial motifs can initiate the inflammasome process by members of the Nod‐like receptors, including the NLRP3 family, which leads to the activation of caspase‐1 [296]. Activated caspase‐1 cleaves pro‐Il‐1β and pro‐IL‐18 into their mature forms, inducing pyroptosis [296]. The activation of this pathway is fundamental in the male bladder cancer urothelial cell line, 5637, for expression of antimicrobial peptide‐related genes [297]. The UPEC strains CFT073 and UTI89 induce inflammasomes in human macrophages, inducing rapid pyroptosis and cell death [298]. This also occurs in mouse bone marrow‐derived macrophages with CFT073 infection and UTI89 at very high multiplicity of infection [298]. The NLRP3 pathway is key for a controlled response to CFT073 in vivo, as its absence increases MMP‐7, which processes Il‐1β to its mature form, causing exacerbated inflammation [299].

### 4.2 Sex Differences in the Detection of Bacteria

Studying the impact of PPR in mice was performed primarily in female mice, limiting our knowledge of these receptors in male UTI. However, one study has compared female C3H/HeJ mice to males challenged with bacteria directly in the kidney by surgery. The male mice succumbed to infection with greater bacterial loads in their bladder and kidney 3 days post‐infection compared to females [300]. As these mice are global knockouts, resident and recruited immune cells will also be deficient, confounding the interpretation of the importance of TLR4 in the urothelium. Indeed, TLR expression on resident and recruited immune cells varies according to sex in other organs, supporting that this could be the case in the bladder, as well. Peritoneal leukocytes express higher levels of TLR2, TLR3, and TLR4, as well as MyD88, in female mice and rats compared to males [301]. Ovariectomy decreases mRNA and protein expression of these TLRs [301]. In the mouse spleen, male F4/80+ macrophages and CD11c + DCs express more TLR2 and TLR4 than female cells [302]. Different TLR expression patterns in resident immune cells may accelerate the formation of a response to UPEC in the bladder.

Since TLR expression on bladder resident immune cells is poorly characterized, we can speculate that expression of these receptors may vary based on sex in the resident compartment. During UTI, myeloid cell subsets, such as eosinophils, neutrophils, and monocytes, infiltrate the bladder [30]. These circulating immune cells have distinct TLR expression according to sex. For example, women have higher percentages of CD16+ monocytes compared to men, and these cells express more TLR2, but TLR4 is not different [303]. Neutrophils from men express higher levels of TLR4 and produce more TNF constitutively [304]. In peripheral blood mononuclear cells, TLR7 is higher in female cells than male cells because the *TLR7* gene, encoded on the X chromosome, escapes X inactivation [305]. Male peritoneal monocyte‐derived macrophages, recruited by incomplete Freund's adjuvant, express higher levels of TLR4 compared to female cells [306]. Freund's adjuvant likely induces increased expression of TLRs at the site of injection [307]. Thioglycolate‐recruited mouse monocyte‐derived peritoneal macrophages have increased TLR2 expression in males at baseline, whereas female monocytes have higher levels of TLR4 [308]. Therefore, TLR expression patterns may differ between the sexes but may also vary based on peritoneal macrophage priming method.

More concretely, expression patterns do not always dictate receptor activity. In a rat model of spontaneous hypertension, increased TLR4 expression does not impact blood pressure or pro‐inflammatory T cells in the kidney between the sexes [309]. However, higher TLR7 levels translate to enhanced responses to TLR7 ligands with more interferon alpha produced from plasmacytoid DCs in female patients in HIV‐1 and systemic lupus erythematosus [310, 311]. In coxsackievirus B3 infection, TLR4 expression remains higher in male DCs and macrophages compared to females 3 and 6 days post‐infection [302]. The same is seen for TLR2 on male DCs, but on macrophages naïve TLR2 expression is decreased compared to females and remains equal to female levels during infection [302]. This may impact infection outcomes as male mice develop more severe myocarditis. Additionally, increased TLR expression on female peritoneal macrophages enhances their phagocytic capacity, an effect which is diminished in ovariectomized mice [301].

Thus, there is a significant gap of knowledge in whether TLR expression is different between the sexes in the bladder urothelium, resident and infiltrating immune cells, as well as during bacterial infection.

## 5 Downstream Transcription Factor Differences Between the Sexes

### 5.1 Sex Differences in Transcription Factor Activity

Downstream of PRR are transcription factors that regulate expression of molecules needed to fight infection. Biological sex impacts diverse points in these downstream signaling pathways: transcription factor expression and the transcription of downstream targets. Of note, most transcription factors are expressed to similar levels between the sexes, but differential activity patterns, or activation, may be induced of the transcription factors [312]. Indeed, the activity of transcription factors may vary between the sexes. In a randomized control trial, women challenged with a low‐dose endotoxin bolus have greater predicted cyclic AMP response element‐binding protein transcription factor activity than men in their peripheral blood mononuclear cells, whereas no differences in NFκB and AP‐1 were observed. Surprisingly, women had higher levels of pro‐inflammatory cytokines, including *TNF, IL8, FOS, JUN*, and *CCL3* [313].

Sex differentially expressed genes are present in 45 non‐reproductive human tissues, the bladder included [314]. The highest differential gene expression was in the brain. In other tissues, including the bladder, these genes are mainly on sex chromosomes or involved in sex steroid production [314]. Approximately 30% of the sex‐differentially expressed autosomal genes in every tissue tested contain estrogen or androgen response elements [315]. Another study shows that the highest sex differentially expressed genes in the brain, thyroid, and breast are not enriched for motifs of the estrogen receptors or androgen receptor [312]. This suggests that mechanisms other than steroid hormone signaling regulate differential gene expression between the sexes; however, these mechanisms remain to be elucidated. Notably, the sex differentially expressed genes are differentially targeted by transcription factors, pointing to a potential regulatory mechanism [312]. This establishes a transcriptionally distinct landscape between the sexes, which may be responsible for differences in tissue homeostasis and response to injury.

In circulating immune cells, the transcriptional profile is also different between the sexes due to a heightened transcription factor response. In plasmacytoid DCs, there are higher levels of ATP‐dependent DEAD‐Box Helicase 3 X‐linked (DDX3X) in females compared to males, suggesting increased capacity to initiate and regulate transcription and translation [316]. This is correlated with increased expression of TLR7 and TLR9, and the TLR‐mediated IFN‐I induction (IRF3 and IRF7) in pDCs isolated from women, suggesting DDX3X is important for regulating female TLR‐mediated immune responses in response to viral stimuli [316].

Specifically, in mouse liver, patterns of differentially expressed genes are associated with distinct chromatin status. Some female‐biased genes are associated with chromatin marks that are similar between the sexes, but in males are repressed by a male‐biased repressor, BCL6 [317]. Other highly female‐biased genes show sex differential chromatin signals and are preferentially activated by the female‐enriched STAT5 binding [317]. This shows there is a fine balance between differentially expressed transcription factors and differential chromatin patterns shaping sex‐specific tissue responses. Chromatin remodeling enhances or decreases gene accessibility for transcription, shaping the transcriptional landscape between the sexes. This suggests that the chromatin accessibility of downstream transcription factor targets plays a role in shaping the immune response in the sexes. Indeed, sex‐specific regulatory patterns of transcription factors are detected in many tissues. In a study of peripheral blood mononuclear cells, over 500 sex‐specific chromatin accessibility regions were identified. Enrichment of sex‐specific open chromatin regions was primarily on the X chromosome, and the top 10% of genes at these open chromatin regions are genes that escape X‐inactivation [318]. Increased gene expression at these sites likely leads to a unique transcriptional landscape in female organisms, not seen in males.

In fact, the sex‐differentially expressed genes in homeostatic human tissues are enriched for methylation, suggesting that epigenetic changes can drive differences in transcription factor activity and sex differences in steady state, but also disease [312]. Differences in chromatin accessibility can be caused by different gene expression due to X inactivation escapee genes, which can include chromatin proteins [319]. In this case, chromatin would be remodeled on autosomal and X chromosomes, causing differential gene accessibility between the sexes [318, 320]. This suggests there can be different chromatin signals in genes between the sexes, and this is possibly different due to different hormone receptor binding.

This opens the question as to whether chromatin landscapes drive differential responses to UTI between the sexes. Urothelial cells from female mice that either resolved infection or developed a chronic UTI have differentially accessible regions of their chromatin [321]. In chronically infected mice, these regions are associated with cell death, oxidative stress, and the immune response, and are enriched for binding motifs of the AP‐1 family of transcription factors [321]. This suggests that female and male mice may have a different chromatin landscape before infection, leading to a faster response to UPEC in female mice.

In conclusion, many differences can be present downstream of PRR activation. There can be differences between the sexes in terms of levels of transcription factors, activation of transcription factors, different targets, or chromatin accessibility, all of which can shape the transcriptional landscape at baseline between the sexes or after a challenge, such as UPEC infection in the bladder. We will explore this in the context of the two main transcription factors downstream of TLR signaling in UTI.

### 5.2 NFκB in the Bladder

NFκB is a well characterized transcription factor associated with the induction of pro‐inflammatory programs. Inactive NFκB complexes are present in the cytoplasm of human urothelial cells from female biopsies [322]. Upon activation by LPS, TNF, or dsRNA, these complexes translocate into the cell nucleus [322]. Intravesical instillation of LPS induces the activation of NFκB in urothelial cells in mice [323]. In female transgenic NFκB‐luciferase Tag mice, treatment with LPS intravenously or chemicals (As_2_O_3_ and CYP) intraperitoneally strongly induces NFκB activity in the bladder, supporting that NFκB is active in this tissue [324]. NFκB activity is also strongly induced after 24 h in female mice with a transgene containing NFκB responsive elements and a lacZ reporter that were intravesically infected with UTI89 [325]. This activity was found in the superficial umbrella cells, showing a positive correlation between this activity and UPEC localizing to these cells [325].

UPEC strain NU14 can inhibit degradation of IκB in vitro in TEU‐2 urothelial cells generated from normal human ureter, which keeps NFκB in the cytoplasm preventing its activation. Although the mechanism of inhibition is unknown, this delay may allow UPEC to establish intracellular communities inside urothelial cells [326]. Whether this mechanism is specific to one sex of urothelial cells is unclear, as the sex of the cells used was not specified. However, this is less likely as the number of UPEC that colonize the bladder at 24 h post‐infection are not different between the sexes in C57Bl/6 mice [30]. In wildtype female mice infected with UTI89, RelA, a subunit of the canonical NFκB pathway that can form homo‐ or heterodimers, translocates exclusively into the nucleus of umbrella cells, peaking as early as one‐hour post‐infection [325]. This process depends on FimH adhesion to umbrella cells and TLR4 engagement [325]. NFκB is functionally active as it can bind to target DNA in the nucleus and increases pro‐inflammatory cytokine expression, including TNF, IL1‐ α, IL1‐β, IL‐12, in vivo [325]. This study was only performed on female mice; thus, whether this occurs in male mice remains to be determined.

Neutrophils isolated from naïve female and male mice release NOX2‐mediated ROS in response to UPEC ex vivo and release the transcription factor nuclear factor erythroid 2‐related factor 2 (Nrf2), in its active state into the nucleus [327]. Nrf2 interacts with the antioxidant response element, which inhibits NFκB signaling, preventing exacerbated inflammation. Infection of NOX2 knockout female and male mice shows decreased levels of Nrf2 but increased levels of NFκB compared to wildtype mice of each sex in whole bladders, suggesting there is a precise balance between pro‐ and anti‐inflammatory responses during UTI [327]. There was no direct comparison between the sexes, and it is still to be determined whether this is a urothelial or immune cell effect in vivo.

Whether NFκB family members are differentially expressed between the sexes at steady state in the bladder is unknown, as in studies of the complex in the cytoplasm of urothelial cells in vitro the sex of the cells is not included, and in vivo, only female mice were used for intravesical instillation of LPS or UPEC [322, 323, 324]. Lending support to the possibility that NFκB family members are differentially expressed in the bladder, in mouse aortic endothelium, TNF‐mediated NFκB signaling is predicted to be greater in the transcriptome of female cells compared to males. This is a predicted difference at baseline, which may determine differences in atherosclerosis between the sexes [328]. Human glutamatergic neurons from brain biopsies have increased TNF‐mediated activation of NFκB in women compared to men, which results in greater protection against neuronal death via oxidative stress. This is accompanied by increased expression of NFκB target genes in female neurons [329]. In female T cells, T cell receptor activation upstream of NFκB leads to Xist RNA localization to the inactive X chromosome [330]. Therefore, NFκB may be responsible for tissue specific functions particularly in female mice, such as the regulation of X‐linked genes that drive or enhance immune responses.

### 5.3 IRF in the Kidney

IRFs can be activated downstream of the TLRs and despite typically having a role in antiviral functions, they also respond to UPEC. IRF7 activation is associated with susceptibility to acute cystitis and disease symptom severity [331]. Polymorphisms in the *IRF3* and *IRF7* promoter are present in children with severe kidney infections and recurrent pyelonephritis [332]. *IRF3* polymorphisms are also present in children who are asymptomatic bacterial carriers [332]. IRF3 and IRF7 can form heterodimers, which activate type I interferons and IFN‐responsive genes [332]. Female mice lacking IRF3 infected with UPEC strain CFT073 develop severe kidney infection with extensive tissue damage and increased neutrophil infiltration. This is accompanied by IRF7 overexpression [333]. Downstream targets of IRF7 include *Junb*, *Stat3*, and *Ikbkg*, which may be responsible for this exaggerated response. Blocking IRF7 abrogates this phenotype in Irf3^−/−^ mice. Interestingly, Irf7^−/−^ mice develop mild UTI with lower bacterial counts than Irf3‐deficient and wildtype mice in the urine and kidneys [333]. In UPEC infection of kidney epithelial cells, ceramides associated with TLR4 on the plasma membrane induce downstream phosphorylation of IRF3 [332]. This shows a tightly regulated interaction between IRF3 and IRF7 is required to drive a self‐limiting innate immune response to pyelonephritis. This has only been shown in the kidney in female mice. It is not known whether the same occurs in infections in the bladders and whether such a tightly regulated process exists in male mice.

Globally, little is known about differential expression of IRF3/7 between the sexes. Female patients with Alzheimer's disease have increased expression of several transcription factors, and notably IRF3 is predicted to be activated with positive enrichment scores in female patients compared to male [334]. Unstimulated immune cells from the ImmGen cell set from female and male mice present enriched pathways for IFN responses (Irf1,7,9) and JAK3‐STAT3 [335]. Finally, in the context of sepsis, 40% of male IRF3 knockout mice survived this challenge, whereas all wildtype male mice died. 80% of the female wildtype mice died, while all the IRF3 knockout female mice survived [336]. This suggests IRF3 or the pathways it regulates act differently between the sexes [336].

### 5.4 Sex Hormone Receptor Signaling

**FIGURE 2:**
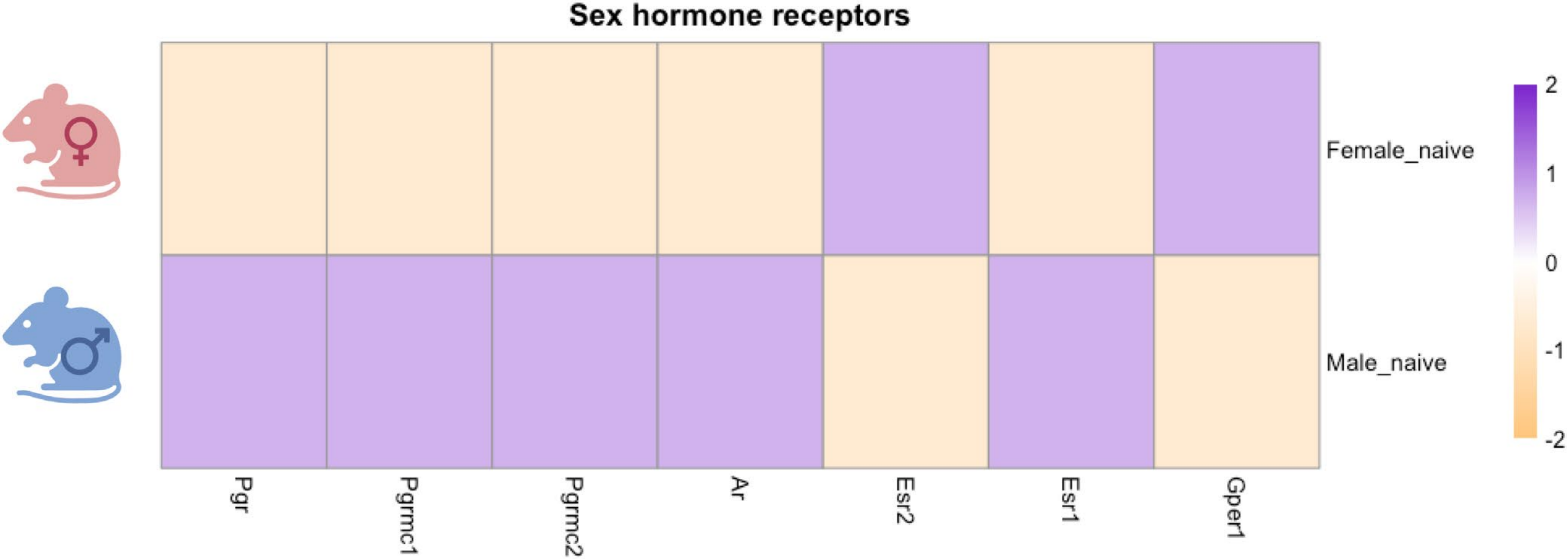
Gene expression of sex hormone receptors in naïve female and male urothelium and resident immune cells located close to the urothelium. Bladders were collected from naïve 6–7‐week‐old C57BL6/J mice. The urothelial layer was separated from the underlying muscle layer [342] and RNA was extracted from this layer for bulk RNA sequencing. The counts obtained were pre‐treated and aligned, then statistically analyzed. Normalized read counts, obtained with DESeq2 package, are shown for the sex hormone receptors found in female and male mouse urothelium. Higher expression is represented in purple and lower expression is orange. Figure was created in R studio (V2025.090.2 + 418) and Biorender.com.

Sex differences in the innate immune response to bacteria can also be a consequence of specific sex hormone signaling. To respond to estrogens, there are two cytoplasmic forms of the estrogen receptor, alpha and beta, which bind diffused estrogen and translocate into the nucleus, as well as guanine nucleotide‐binding protein‐coupled estrogen receptor 1 (GPER1) on the plasma membrane [337]. In humans, the expression of sex steroid receptors varies depending on the organ, with higher expression of ERα in the uterus, ovaries, and breast, and ERβ in the nervous system, cardiovascular system, gastrointestinal, and male reproductive tract [338]. Although surprisingly not widely studied, the expression of sex steroid receptors can vary between the sexes; for example, there are increased transcripts of ERα and GPER1 in female rat kidneys [339]. However, not only are sex steroid hormone levels different between the sexes, contributing to different levels of signaling, but once in the nucleus, the receptors exhibit sex differential regulatory targeting patterns, especially in the breast, heart, and whole blood [19, 312]. This will also contribute to diverse responses between the sexes. Sex steroid hormone receptors are widely expressed by immune cells in circulation, as well as epithelial cells, but it is not specified whether this is different between the sexes [113, 340, 341]. We observed that the urothelium expressed transcripts of sex hormone receptors that differ between naïve female and male mice (unpublished data, Figure 2). We found that Esr1 and Gper1 were more highly expressed in naïve female urothelium, whereas the androgen receptor and progesterone receptors were found in greater quantities in naïve male urothelium. Thus, the activity of the estrogen and androgen receptors may differentially modulate TLR‐mediated innate immune responses in the bladder.

ERα contributes to enhanced cytokine responses downstream of TLR. In ERα knockout female mice, splenocytes, B cells, and kidney epithelial cells stimulated with TLR 3, 4, 7, and 9 agonists show reduced cytokine production [343]. In mouse bone marrow‐derived conventional DCs, ERα increases activation of the IL‐23‐IL‐17 axis [343]. Since the effects are not specific to a single type of TLR, it is possible the same effect occurs in female organisms or is hindered in males in UTI, as an IL‐17‐mediated immune response is necessary for resolution of infection in female mice, but is lacking in male mice [30]. Inflammatory cues, such as lipoteichoic acid activating TLR2 and Il1β, can induce the methylation of ERα. The methylated receptor remains cytoplasmic and binds to MyD88 in male monocytes (THP‐1) and female kidney epithelial‐like cells (HEK293T), inducing NFκB activity, but interestingly, in the presence of estradiol, the binding to MyD88 is abrogated [344]. This suggests this interaction occurs endogenously and ERα contributes to the pro‐inflammatory TLR response.

Activation of GPER1, with specific agonists or 17β‐estradiol, leads to a decrease in TLR4 expression of male RAW264.7 macrophages [345]. In vivo, female mice treated with 17β‐estradiol have increased TLR4 and CD14 expression in serum macrophages and peritoneal macrophages [346]. However, this increases the susceptibility of female mice to endotoxin with higher levels of TNF and disease severity [346]. By contrast, human primary monocytes of both sexes treated with 17β‐estradiol activate GPER1, but also the non‐classical 36 kDa splice variant of ERα, which inhibits the response against LPS challenge [347]. The NFκB pathway is blocked by ERα‐36‐kDa binding to p65, preventing the secretion of IL‐6, and GPER1 acts as a coregulator of this process [347]. Additionally. ERα‐36‐kDa signaling leads to decreased cytokine production in female mice with influenza treated with estradiol [348].

Finally, androgens acting via the AR have an overall suppressive effect on the immune response. In several mouse cancer models, AR signaling leads to expression of ubiquitin specific peptidase 18, which inhibits TAK1 phosphorylation, inhibiting the activation of NFκB. Therefore, male mice have reduced anti‐tumor immunity [349]. Testosterone decreases the expression of TLR4 on male RAW 264.7 macrophages and on male mouse primary macrophages from mice with no endogenous androgen production [350]. In vivo TLR4 expression is increased in male peritoneal macrophages and monocytes in the absence of endogenous testosterone, and this worsens the severity of endotoxic shock [350]. Additionally, TLR4 is upregulated in the prostate after castration [351]. Since prostatitis possibly acts as a bacterial reservoir contributing to the chronicity of UTI in male mice, this may be due to the decreased levels of TLR4, leading to attenuated responses.

Evaluating the effects of sex hormone signaling on the TLR pathway is challenging many studies only focus on cell lines of male origin (THP1, RAW 264.7, HEK293T) and transcriptional activation of the receptors differs between the sexes [312]. Sex steroid hormone signaling can have both pro‐ and anti‐inflammatory effects depending on the receptor activated, the isoform involved, physiological or supraphysiological levels of hormones present, and differences in the stimuli (e.g., autoimmune, cancer, viral, bacterial). Therefore, there is a fine‐tuning of the TLR response, which may already be different between the sexes, and the intricate mechanism of hormone signaling may impact the initiation of a TLR‐mediated innate response.

Indeed, in our research, sex hormones impact the outcome of infection between the sexes. Testosterone, and androgens more generally, may have an imprinting effect on immunity, which impacts bacterial clearance, as testosterone‐treated female mice, male mice, and estradiol‐treated castrated male mice are unable to clear infection [30]. In vitro, the treatment of male urothelial cells with estradiol did not impact the colonization of CFT703, but it did boost the NLRP3 response leading to increased antimicrobial peptide expression [297]. ERβ protein is expressed in the bladder of women, and thus could influence UTI [352]. Post‐menopausal women taking estrogen have a reduction in UTI recurrence [353, 354]. Therefore, it is possible that estrogens and androgens impact the initiation of the innate TLR‐mediated response between the sexes, leading to a rapid and efficient immune response in the female mice but not in male mice.

## 6 Concluding Remarks

Bacteria need to overcome several layers of host defense to invade and colonize the epithelium of a mucosal tissue. Sex differences exist in the incidence of infection, host immune response, and progression of infection, with UTI being a prime example with greater incidence in women and stronger immune responses and better outcomes in female mice, mirroring the disease in humans. Sex differences in the defense mechanisms bacteria, such as UPEC, must overcome may include the mucus of an organ, which they need to traverse to reach epithelial cells. Despite numerous fundamental studies of the mucus in the gastrointestinal and respiratory tract, there is limited information on how this layer may be different between the sexes, including structural and biochemical differences that could impact bacterial interactions and infection. Once bacteria reach an epithelium, they must bind and potentially invade these cells to colonize the tissue. Differences in surface receptors or other factors used by the bacteria may be divergent between the sexes. Finally, it is crucial for the host to mount a rapid immune response, and this is usually governed by the epithelium and resident immune cells. Whether differences between the sexes at the level of PRR expression, downstream activation, transcriptional patterns, and influence of sex hormone receptors exist is still poorly defined in many mucosal sites. These factors will shape how effectively and rapidly the host will initiate an innate response against UPEC and may be responsible for the attenuated immune response observed in male mice leading to chronic infections. Researching these initial host‐pathogen interactions will allow for further understanding of UTI in both sexes and in turn, improve treatment for the disease that has different outcomes in women and men.

## Author Contributions

L.R.F. and M.A.I. wrote and edited the manuscript. Generative AI was not used in the writing or editing of this manuscript.

## Funding

This work was supported by Agence Nationale de la Recherche, ANR‐19‐CE15‐0015, ANR‐21‐CE15‐0006, ANR‐22‐CE15‐0022. Pasteur‐Paris University (PPU) International PhD program.

## Conflicts of Interest

The authors declare no conflicts of interest.

## Data Availability

The data that support the findings of this study are available from the corresponding author upon reasonable request.
